# Uncertainties experienced by nursing professionals who contracted Covid-19: a priori, everyone is human

**DOI:** 10.1590/0034-7167-2023-0142

**Published:** 2024-06-28

**Authors:** Cinthia Rafaela Amaro Gonçalves Andrade, Laís de Miranda Crispim Costa, Ingrid Martins Leite Lúcio, Janaina Ferro Pereira, Herika do Nascimento Lima, Renata Lira do Nascimento

**Affiliations:** I Universidade Federal de Alagoas. Maceió, Alagoas, Brazil

**Keywords:** Nursing, Nursing Theory, Nurse Practitioners, COVID-19, Coronavirus Infections, Enfermaría, Teoría de Enfermaría, Enfermeras Practicantes, COVID-19, Infecciones por Coronavirus

## Abstract

**Objective:**

To analyze the uncertainties experienced by nursing professionals who contracted COVID-19.

**Methods:**

This qualitative research was conducted with 20 nursing professionals who fell ill from COVID-19. Data collection was carried out through semi-structured interviews; the data were then organized using thematic analysis and discussed in the context of Merle Mishel’s Reconceptualized of Uncertainty in Illness Theory.

**Results:**

The antecedents of the disease had a strong influence on how nursing professionals who contracted COVID-19 perceived uncertainty. The media coverage of the increasing number of cases, the collapse of the healthcare system, and the high mortality rate contributed to associating the disease with fear and panic.

**Final Considerations:**

Viewing it from the perspective of the disease’s antecedents, the illness of a nursing professional from COVID-19 underscores that before being professionals, they are human beings just like anyone else, undergoing adversities and facing the possibilities associated with being ill.

## RESUMEN

**Objetivo:**

Analizar las incertidumbres experimentadas por profesionales de enfermería que enfermaron por COVID-19.

**Métodos:**

Investigación cualitativa realizada con 20 profesionales de enfermería que enfermaron por COVID-19. La recolección de datos se llevó a cabo mediante entrevista semiestructurada; los datos se organizaron a partir del análisis temático y se discutieron a la luz de la Teoría Reconceptualizada de la Incertidumbre en la Enfermedad de Merle Mishel.

**Resultados:**

Los antecedentes de la enfermedad tuvieron una fuerte influencia en la apreciación de la incertidumbre de los profesionales de enfermería que enfermaron por COVID-19. La difusión mediática del aumento de casos, el colapso del sistema de salud y el alto número de muertes contribuyeron a que la percepción de la enfermedad se asociara con el miedo y el pánico.

**Consideraciones Finales:**

Desde la perspectiva de los antecedentes de la enfermedad, el padecimiento de los profesionales de enfermería por COVID-19 mostró que, antes de ser profesionales, son seres humanos como cualquier otro, enfrentando adversidades y potencialidades de personas enfermas.

## INTRODUCTION

This study focuses on the experiences of uncertainty faced by nursing professionals during their illness process from Covid-19. Caused by the coronavirus SARS-CoV-2, Covid-19 is an acute respiratory disease, potentially severe in humans, and is characterized by high rates of contamination and global spread^(1)^. Knowledge about the transmission of Covid-19 is continuously updated. Currently, it is known that the incubation period is estimated to be between 1 and 14 days, with a median of 5 to 6 days. The primary mode of transmission is person-to-person through respiratory droplets. Most transmissions occur from symptomatic individuals to others; however, studies indicate that approximately 45% of all transmissions occur before the onset of symptoms^(2)^.

The Covid-19 pandemic and its repercussions extend far beyond the biological realm and the technical race of science in search of control measures, treatments, vaccines, and knowledge production to control the virus’s spread. It also has significant impacts in economic, psychological, and social dimensions, affecting health determinants and conditions, not just for the ill but for the general population as well^(3)^.

“The pandemic consists of countless outbreaks and epidemics, individual and family suffering, similar in biological aspects but incomparable in others”^(4)^. Therefore, this study is significant for its scientific contributions concerning the illness of nursing professionals affected by Covid-19, considering the unique knowledge in this field.

## OBJECTIVE

To analyze the uncertainties experienced by nursing professionals who contracted Covid-19.

## METHODS

### Ethical Aspects

The study was conducted in accordance with both national and international ethical guidelines and was approved by the Research Ethics Committee of the Federal University of Alagoas. The committee’s opinion was attached during the submission process. Before the interviews, participants read and signed an Informed Consent Form. To ensure anonymity, participants were assigned abbreviated codes: “ENF” for nurses and “TEC ENF” for nursing technicians. The alphanumeric coding combined the professional category with the sequence number of the conducted interviews.

The guidelines and ethical standards set forth in Resolution 466/12 and No. 510/2016 of the National Health Council regarding research involving human beings were adhered to.

### Type of Study

This was an exploratory-descriptive study with a qualitative approach. The COREQ (Consolidated Criteria for Reporting Qualitative Research) instrument was utilized.

### Study Setting

The study was conducted in the state of Alagoas, located in the eastern part of Northeastern Brazil. Alagoas encompasses 102 municipalities and covers an area of 27,778.506 km^2^, bordering the states of Sergipe, Bahia, Pernambuco, and the Atlantic Ocean.

### Data Source

Twenty nursing professionals participated in the study, providing testimony through semi-structured interviews. Inclusion criteria were nursing professionals with a confirmed Covid-19 diagnosis, residing in the state of Alagoas from October 2020 to May 2021. The participants were characterized as follows: 16 nurses and 4 nursing technicians, ranging in age from 27 to 46 years, with only 2 being male. In terms of marital status, 16 were married, 3 divorced, and 1 widowed. The most commonly self-reported skin color was brown (14), followed by white (5), and black (1). The participants had high workloads, with 11 reporting 100 hours/week and holding multiple job contracts.

The confidentiality of the study was maintained as there was no public disclosure of results that could lead to the identification of the participants, thanks to the use of alphanumeric coding.

### Data Collection and Organization

After receiving approval from the Brazil Platform, the research team initiated contact with participants, beginning with three nursing professionals who had contracted Covid-19 and were known to the researchers. Each of these professionals then referred another individual who met the inclusion criteria, and this process continued. Data collection concluded upon reaching data saturation^(5)^, a point at which additional data and information do not significantly alter the understanding of the phenomenon under study. Participants were approached either in person or by phone, where the researcher presented the study proposal and, upon agreement, scheduled an interview. Data collection took place between January and July 2021.

The interviews, led by the principal researcher, lasted approximately 20 minutes each. They were conducted in a private location, agreed upon beforehand with each participant. The sessions were recorded using a digital recorder, and the content of the interviews was transcribed verbatim into a Microsoft Word document for subsequent analysis.

### Data Analysis

The thematic analysis was conducted following Minayo’s methodology^(5)^, which comprises three stages: 1) pre-analysis, involving initial reading, corpus formation, and the (re)formulation of hypotheses and objectives; 2) exploration of the material; and 3) processing and interpretation of the results obtained. Merle Mishel’s Reconceptualized of Uncertainty in Illness Theory (RUIT)^(6)^ served as the theoretical framework.

The theory asserts that uncertainties are present in all stages of illness (before, during, and after). This idea is built upon three foundational pillars of the theory: 1) Antecedents of the disease; 2) Cognitive appraisal of uncertainty; and 3) Coping strategies^(6)^. These categories guided the selection of participant narratives presented in the results. Additionally, according to the theory, individuals may recognize beneficial aspects of uncertainty throughout the course of the disease, leading them to value each moment of their lives, a phenomenon Mishel refers to as growth through uncertainty^(7)^.

## RESULTS

As a novel disease, Covid-19 emerged as one of the greatest global health challenges of this century. Some statements illustrate how this reality was previously unknown:

[...] *at the beginning of the pandemic, our experience in Brazil was a bit scary. It wasn’t easy to see people in Ecuador being carried away in trucks. Bodies were being transported, and in some places, there was no more room for burial.* (ENF 01)

[...] *the situation was already critical in some countries, and I couldn’t imagine it would become so devastating in Brazil. It was a time when everything was new and uncertain.* (ENF 03)

[...] *the onset of the pandemic in March 2020 brought a series of uncertainties for everyone. We didn’t know what we were going to face.* (ENF 09)

[...] *it was a new challenge, a pandemic, a virus that no one knew about, that no one thought could happen.* (ENF 18)

[...] *actually, when people started talking about Covid, I wasn’t very interested because I thought it wouldn’t reach Brazil.* (ENF 10)

[...] *although Covid was already being discussed, I didn’t believe it would affect us here.* (ENF 20)

[...] *when I watched it on TV, it seemed unreal. It becomes real when it comes close to you, like at your workplace. Then you realize, my God, it’s real. I only started to grasp the situation when it reached us. Before that, it felt unreal*. (ENF 02)

Analyzing the overall context, Covid-19 is a phenomenon of significant magnitude and relevance, with information disseminated through social media and journalism:

[...] *I was quite worried after seeing the severity of the disease in humans reported by the media, and it deeply affected me.* (ENF 09)

[...] *before the disease reached Brazil, I watched a lot of news. Seeing the reports on TV, especially about Italy, Spain, and other wealthy countries with many deaths, made me nervous.* (TEC ENF 04)

[...] *so I really tried to avoid watching the news. The constant reports of death were overwhelming.* (ENF 03)

They also noted that at the beginning of the pandemic, there was a lack of information and standardized clinical management protocols based on scientific evidence, making clear judgments difficult:

[...] *being a very new disease, there was a lot of conflicting information.* (ENF 14)

[...] *at that time, there was limited knowledge about Covid-19; it was something new.* (ENF 20)

[...] *I only had information from other countries. I knew that many places were shutting down and that isolation was the recommended treatment.* (ENF 03)

[...] *then came the ‘boom’, the use of masks, as it was understood to be an airborne disease. At the time I became ill, that was my understanding.* (ENF 11)

At the beginning of the pandemic, as health information was still being processed and developed, the interviewees noted that the constant changes in information hindered its assimilation and exposed them to a vulnerability in knowledge:

[...] *I don’t think I can consider as knowledge what was being broadcast in the news at that time because the information about what was happening around the world was very specific, and it still hadn’t been defined.* (ENF 01)

[...] *it came to us with so much uncertainty, we didn’t know the extent or the nature of it. It reached an extreme.* (ENF 02)

[...] *in the beginning, as it was a new disease, there were many conflicting pieces of information.* (ENF 09)

Thus, the context of insecurity due to a new disease, with its conflicting information, fostered astonishment and fear about the possibility of falling ill, generating uncertainties even before the event occurred. In these circumstances, it is emphasized that being a healthcare professional does not negate one’s essence as a “Human Being”, a “Subject”.

[...] *what the TV showed were the severe cases, and I was very afraid of acquiring severe acute respiratory syndrome. The fear was more about the disease itself.* (ENF 10)

[...] *the truth was, you only saw the cases increasing, people getting worse, everyone catching it. Waiting anxiously for your turn.* (ENF 16)

[...] *you couldn’t touch anything, not money, tables, or counters; everywhere there was a risk of catching the disease.* (ENF 03)

[...] *having to stay away from everything, especially work, considering my active and dynamic nature, was also difficult.* (ENF 06)

In line with this, nursing professionals who contracted Covid-19 at the beginning of the pandemic experienced a lack of clarity about the disease due to the scarcity of information.

[...] *my understanding of Covid was that it was a highly contagious respiratory disease, with initial symptoms of fever and weakness, and that was all I knew then.* (ENF 11)

[...] *up until that moment, I knew it was serious, but I didn’t have much knowledge about it, mainly due to the overall lack of information.* (TEC ENF 07)

[...] *what causes a virus to become so severe from such mild initial symptoms, and why does it vary so much from person to person? Some people manage it well, while others end up dying.* (ENF 08)

[...] *‘Do you know everything about it?’ No! I don’t, because it changes every day! Just yesterday, I watched a live stream with a cardiologist and an infectious disease specialist, and they shared information that left me more confused than before! Every day brings something new!* (ENF 12)

At times, scientific research was crucial for a better understanding of the disease, its effects, and the search for solutions, as observed in the following statements:

[...] *I remember that many manuals went through various revisions, so we were always keeping up with this dynamic process. I believe that absorbing knowledge from the Ministry of Health coincided with the process being developed at the health department.* (ENF 01)

[...] *in some ways, researchers are enhancing the scientific evidence about the disease every day, improving the information for health professionals and the general public.* (ENF 09)

[...] *the level of knowledge, information, and treatment we have evolved since 2020 has been very important. The knowledge is gradually becoming more consolidated.* (ENF 14)

[...] *even without comprehensive knowledge, we already understand that people in high-risk groups are more prone to severe complications.* (ENF 19)

However, with the appearance of cases outside the profile of high-risk groups, fear intensified:

[...] *today’s reality shows that there is no specific risk group, as mortality is occurring from newborns to the elderly. This has dispelled the belief that only those with serious diseases or comorbidities are at risk.* (ENF 01)

[...] *we know of people with extensive knowledge who are taking all precautions, have a healthy lifestyle in terms of diet, personal care, and who are not sedentary, yet are developing very serious cases and even dying, contradicting initial beliefs.* (ENF 08)

[...] *each person reacts differently. It depends on the individual’s body. For example, some may have an oxygen saturation of 97%, which suddenly drops to 93%. Others might maintain a saturation of 97, 98 and experience shortness of breath and fatigue, an intense sleepiness.* (ENF 13)

[...] *I recently lost many loved ones, including a 48-year-old cousin two weeks ago who had no comorbidities and was an active cyclist, but unfortunately, he did not have the same luck I did. We will never forget this difficult part of losing loved ones.* (TEC ENF 15)

[...] *we already knew it was a serious disease, and everyone was at risk.* (TEC ENF 05)

Participants also reported fear of contracting Covid-19, expressing insecurity regarding a potential diagnosis:

[...] the real discomfort is that we don’t know what will happen, which creates insecurity. We feel insecure because nothing is certain, there’s no definitive answer, right?! (ENF 08).

[...] today, even with vaccination and having been vaccinated and contracted the disease, the fear in me is persistent. We know that this threat is still new, and there is a long journey ahead to ensure that a medication can help fight the virus in the human body (ENF 09).

In this context, the fear of dying from COVID-19 affected some participants:

[...] *the knowledge we had was something everyone thought might not be correct because it was a disease where, in the end, you thought, ‘everyone is going to catch it and everyone is going to die, and there will be no one left in the world’.* (ENF 16)

[...] *being on the front line, I spent a lot of time caring for patients with fifty, seventy-five, and eighty percent lung impairment, so I was scared. It was the first time in my life I was afraid of dying.* (ENF 13)

[...] *it was not anxiety... it was fear, because I had already had a heart attack, so I was afraid of dying.* (ENF 12)

The fear of contamination led to the implementation of preventive hygiene measures, personal protection, and cleaning routines in line with World Health Organization guidelines:

[...] *we learned that we need to improve our hygiene habits and our respiratory etiquette, which we talked so much about this year, right?* (ENF 01)

[...] *I used the tools I had against the Coronavirus. Since the beginning of the pandemic, I have practiced social distancing, worn masks, and frequently used hand sanitizer to try to keep the disease at bay.* (ENF 09)

[...] *so it was mostly about taking precautions, washing hands a lot, using hand sanitizer, avoiding leaning on furniture.* (TEC ENF 05)

[...] *but the importance of changing clothes when getting home, avoiding entering through the main door and having an alternative entrance, these are some of the precautions we took and learned to deal with this specific disease.* (ENF 02)

[...] *what could we do!? We maintained social distancing and didn’t allow visitors at home. It was our choice, especially when the babies were born, not to receive these visits from family and relatives.* (ENF 14)

In this context, the influence of the disease’s antecedents, such as working on the front lines against Covid-19, illnesses of family members, friends, and close acquaintances, as well as previous health conditions, contributes to uncertainty through probabilistic thinking and the search for predictability of events:

[...] *so, I was diagnosed with Covid in June 2021. But I started showing some symptoms in early May, which is strange considering the time gap to say that I fell ill with Covid in May.* (ENF 19)

[...] *I think when you’re in healthcare, you already have an idea of what might happen to us.* (TEC ENF 05)

[...] *precisely because we are in the field, we are conscious of the severity of the disease.* (ENF 17)

[...] *one day, my 83-year-old mother said to me, ‘My throat is scratchy.’ I wondered how her throat could be scratchy. Then we became worried. Everything could be Covid, right!?* (ENF 20)

[...] *I think it’s even worse for us because not only do we know, but we also experience the day-to-day of patients who contract it and become severely ill.* (ENF 16)

Social support from family is among the factors that help individuals cope with uncertainty. Yet, some professionals chose to distance themselves from their families while working on the front lines against Covid-19 as a form of protection:

[...] *so from the moment I decided to work with Covid, I left my home and avoided my family for a few months.* (ENF 19)

[...] *when I began working directly with Covid patients, I decided to leave home to protect my family since they were practicing social distancing. Therefore, I preferred to leave and keep them safe, which turned out to be beneficial.* (ENF 03)

[...] *it’s a personal matter; I have elderly people in my family, so I was careful not to visit or gather with them. We knew that being in a hospital environment, we could transmit it to more vulnerable family members. So, I took this precaution and still do. However, it’s not easy.* (ENF 13)

Changes in daily interpersonal relationships after the emergence of Covid-19 were marked by a reliance on digital and telecommunication resources, which weakened family and friendship bonds:

[...] *I spent more than five months unable to visit my mother, niece, brother, and my dog. This situation evoked a feeling of helplessness about how much we humans depend on affection, love, and care* [...] *speaking over the phone will never be the same as being in person. Sending a photo by phone will never replace a hug, a kiss on the cheek, the affection of your dog, or meeting childhood friends in the place where you grew up.* (ENF 09)

Previous experiences with illness leading to worsened conditions were also presented as antecedents of the disease, raising uncertainties before contracting the illness due to the association with prior exacerbations:

[...] *being hospitalized resurrected the ghosts of my childhood, especially since my brother was hospitalized for more than 25 days in a hospital bed to treat Covid-19, with over 50% of his lungs compromised.* (ENF 09)

Regarding the life experiences of participants, events and memories linked to relatives and close friends were mentioned, fostering familiarity with the facts:

[...] *I recall that during this Pandemic, a friend’s mother passed away, and it wasn’t easy for him. We were very reflective about it, imagining ourselves in such situations. Imagining the illness of loved ones and the loss of those we love. Being the vector of the disease.* (ENF 01)

[...] *the next day, my brother’s condition worsened, and he was admitted to the hospital. He recovered, and even from his hospital bed, he urged me to take even more care because the disease is challenging. I felt helpless when my brother and cousin were hospitalized, the same feeling I had when my father was hospitalized and passed away months later.* (ENF 09)

[...] *thankfully, my symptoms were mild, but, for example, I had an aunt who was hospitalized for fourteen days. She didn’t require intubation or anything but needed oxygen. So, we try to pass on this guidance to family and close friends, who are always seeking advice on what to do.* (ENF 14)

Thus, nursing professionals use the experiences of their social circle as a resource for interpreting stimuli of uncertainty.

[...] *but when it arrived here, seeing people and hearing things like, ‘So-and-so is going to die!’ right in front of you* [...] *the situation worsening. Colleagues in the Intensive Care Unit* [ICU]*. Colleagues who haven’t been able to leave yet, who are in SARA trying to recover, walk, and speak properly. All this affects us. It’s complicated.* (ENF 02)

[...] *I know that even after witnessing all this, in both of my roles, I remember well that I didn’t freak out. But I had friends who were hospitalized, friends who lost family members, and inevitably, we get affected, but I didn’t freak out to the extent of saying so. I was able to work with balance.* (ENF 03)

[...] *I had colleagues, physiotherapists, doctors, nurses, technicians, become seriously ill, and we were in that frenzy of ‘my God, my God, they have to get out, they have to get well,’ and it was a daily struggle. It got much worse; I think we became more anxious, more nervous, more thoughtful because of the day-to-day experiences.* (ENF 16)

Antecedents of weakened mental health are identified as factors that intensify the condition of fragility.

[...] *I needed help. I needed to take a break. I had to take five months off, and I needed to help myself.* (ENF 02)

[...] *I think it was more psychological, due to the heart attack. But I also had a panic attack.* (ENF 12)

On the front lines in the fight against the new coronavirus, nursing professionals, like doctors and many other professionals, faced a scenario of dual uncertainties, both in providing care and in their own health. This caused stress and concern in their routines:

[...] *I work in an ICU, so it’s a time when you need to have strong nerves. Why? Because you need technical skills to act, to work, and you need to be quick. Not everyone has these characteristics. So what I often saw happening, time and again, was someone getting soiled with secretions or blood, or tracheal secretions, whatever it may be, thinking they were contaminated and going to get sick, but no! You need to stay calm.* (ENF 01)

[...] *it began with patients deteriorating quickly. Patients going into cardiac arrest. Patients dying in a short amount of time. For example, a patient is admitted to a hospital today and within three to four days, they’re intubated. Soon the patient starts using all available vasoactive drugs to keep them alive. Then a complication arises. The patient needs dialysis. And suddenly, the patient passes away. A week, sometimes less. Very quickly.* (ENF 13)

[...] *because, you see, I have a friend at work, not here, I look and respect, but she comes in, puts a plastic on the bed where she rests, sprays Lysoform* [a bactericidal cleaning product] *on the bed, and sleeps in a bag. She rests wrapped in a bag. You see that she has reached the limit of stress. This is the stress limit.* (ENF 02)

## DISCUSSION

In this study, we highlight the significant presence of reports associated with experiences prior to nursing professionals contracting Covid-19. It is observed that these experiences directly influence the appraisal of uncertainty. The research reveals that in the antecedents of uncertainty, we find encouragement, stimulation, and other such adjectives that boost cognitive capacities and mechanisms functioning as sources of stimulus for uncertainty.

It should be noted that the pandemic context brought about peculiarities in the illness process, as mass contamination of the population was expected. Among those who fell ill were health professionals, especially nurses, who, as human beings, have their own life histories and perceptions of the world around them. Thus, new experiences are evaluated through the lens of prior experiences.

According to Mishel, the antecedents of the disease confer or enable the individual to initiate the process of appraisal and decision-making. In experiencing illness from Covid-19, the starting point disrupts this experience, as the fragility experienced conditions cognitive capacity and, consequently, the processing of information^(6)^.

In nursing care for those ill with Covid-19, working on the front lines also has implications in the realm of feelings, including loneliness, helplessness, physical fatigue, stress, irritability, nervousness, and mental exhaustion. It is important to highlight that such feelings negatively impact human relationships and social practices of individuals, especially in caregiving. Thus, ‘they can compromise the capacity to make decisions due to fear, inability to face suffering, and lack of knowledge’ ^(8)^.

This set of elements demonstrates that the pandemic scenario was enveloped in uncertainties, with individual repercussions filled with feelings, challenges, difficulties, images, and behaviors unique to each person. Therefore, scientific evidence suggests that antecedents are fundamental for understanding individual experiences in illness situations, as these can affect the understanding of the illness process, the outcomes of disease progression, and adaptive responses ^(6-9)^.

The Disease Antecedent component, as presented by Merle Mishel in her RUIT, can be elucidated by discussing what each person brings to the experience of uncertainty in the health-illness transition process, influencing how the individual embraces and deals with uncertainty. In the antecedents of the disease, we find motivation, cognitive capacities, and mechanisms that function as stimuli for uncertainty ^(6-7)^.

In the studied context, the media also played a significant role in influencing the degree of uncertainty generated, acting as a source of stimulus for antecedent uncertainty, leading to a negative effect of threat due to the high impact of reported information. In situations where people deal with limited knowledge or a lack of understanding of what they are experiencing, they often exhibit behavior of seeking information that is unstable and/or inconsistent ^(6-9)^, given that scientific evidence for clinical management and related areas was still in the process of being established and consolidated.

From the reports, there is a generalized discourse of people, even professionals in the field, seeking basic health knowledge. Therapeutic progress and practical aspects of dealing with daily and continuous self-care demands are achievable with the appraisal of health conditions and associated vulnerabilities ^(6-9)^. There was an extreme fear of contracting the infection ^(10)^, with psychological symptoms in health professionals being comparable to those seen in other outbreaks, such as SARS and Ebola ^(11)^.

From the perspective of the RUIT, uncertainties in illness experiences encompass ideas of fragility in information, doubts, probabilities, incapacities, discomfort, and insecurity in decision-making, along with difficult sensations and feelings of control and self-confidence. The uniqueness of individual responses is thus explained based on previous experiences and life events, defining future possibilities of existence ^(12)^.

It was noted that uncertainties manifest as inadequacies in the cognitive state, impairing the interpretation and evaluation of disease experiences and compromising the adaptation of the ill person. According to Mishel, a lack of information occurs when sharing information about the disease is absent or insufficient, necessitating additional instruction for the construction of grounded knowledge ^(6-7)^.

As information was validated, it was updated and published dynamically at short intervals. Insecurities can be alleviated by providing information about the causes and consequences of symptoms. In the context of Covid-19, professionals lacked knowledge about the cause, and care for the ill was not yet a part of their work routine ^(13)^.

Cognitive capacities are attributes that individuals possess for verifying certain data and information. This capacity is demonstrated through both innate and specific responses to certain situations. Inference refers to the evaluation of uncertainty, using past experiences to process current experiences and establishing familiarity with the facts. It involves the judgment an individual makes based on lived experiences ^(6-7)^.

A qualitative study with 21 nurses who worked in the emergency or intensive care unit of a hospital in Turkey during the pandemic showed that despite experiencing multiple sources of uncertainty, these professionals focused on patient care and satisfaction as a way to manage uncertainty and give meaning to their experience, recognizing their personal and professional growth throughout the process ^(14)^.

It is important to note that the atypical nature of the pandemic context meant that the antecedents of the disease were already shrouded in uncertainties and part of the illness process due to the fear of affliction. This, combined with belonging to a high-risk group with a greater propensity for health complications, heightened the fear. For participants in the more vulnerable age group for Covid-19 complications, anxiety was more pronounced. According to Mishel, the inability to control the situation leads to difficulty in organizing what is known about the disease, fostering a feeling of insecurity ^(6-7)^.

The insufficiency in valuing the illness process is directly related to uncertainty. Studies indicate that the onset of uncertainty in illness leads to disordered feelings and attitudes, causing individuals to distance themselves from their social circle, generating despair, lack of self-recognition, and changes in personality. Therefore, the theory emphasizes the imperative need for the reorganization and restructuring of coping strategies in response to the manifestation of uncertainty ^(6-7)^.

Nursing workers involved in caring for Covid-19 patients significantly exposed themselves to the risk of illness, with the heterogeneity of this workforce contingent leading to different forms of exposure, both to the risk of contamination and to factors associated with working conditions ^(15)^.

Although healthcare professionals are accustomed to dealing with psychological tension at work, the Covid-19 pandemic presented a different scenario, as they faced the fear of becoming infected and exposing their families to risk. Based on previous experience, the antecedents of the disease allow for a probabilistic analysis of illness, judging it as either a threat or an opportunity. These antecedents condition the degree of adaptation to the familiarity with the facts and to previous experiences, more clearly evidenced in the statements of professionals who worked on the front lines caring for people affected by Covid-19.

Thus, professionals involved in the pandemic response experienced a wide range of case evolutions (mild, moderate, severe, and critical) and prognoses, interpreting the meaning of illness from their own experiences. They made associations between patients’ signs and symptoms in clinical practice during their professional activities to establish similarity and familiarity with health conditions, seeking tangible support and certainty to consolidate knowledge about the disease.

According to Merle Mishel, the reduction of uncertainty is directly linked to structure providers. These help individuals determine the pattern of symptoms, the relationship between events, and the congruence of experiences. As the pattern of symptoms increases, these three aspects also rise ^(6-7)^.

It is clear that social and cultural changes, resulting from the death and illness of emotionally close individuals, trigger the need for restructuring positions, roles, and functions within the family nucleus ^(6-7)^. Reports of memories and events of Covid-19 illness among family members and close acquaintances were quite evident.

This aspect is part of the antecedents of the disease, associated with stimuli related to the disease and physiological issues such as frequency, intensity, quantity, location, and duration; and to the cognitive capacity related to personal information processing skills ^(6-9)^. Memory events with colleagues were also highlighted, as when associated with illness from Covid-19, which already brings many uncertainties, they amplify anguish, despair in healing, and create obstacles in coping strategies for optimizing adaptive responses.

Healthcare professionals, due to the nature of their work, are exposed to a high workload and are part of the high-risk group for contracting Covid-19 ^(15)^. They have faced situations of pressure, risk of infection, excessive work, frustration, discrimination, isolation, care for patients with negative emotions, lack of contact with family, and exhaustion.

These situations lead to mental health problems such as stress, anxiety, depressive symptoms, insomnia, denial, anger, and fear. These issues affect the performance of professionals and also contribute to uncertainties when afflicted with illness from Covid-19. The events that cause uncertainty can be the greatest source of stress, triggering physiological reactions and heightened emotions ^(6-7)^.

### Study Limitations

This study has certain limitations that should be considered in interpreting the results. Firstly, the qualitative nature of the research limits the generalization of the conclusions to the entire population of nursing professionals in Brazil, as experiences and perceptions are inherently subjective and specific to the context of the participants. Another significant limitation is the potential for selection bias, as the participants were chosen through a chain sampling approach, which may have influenced the homogeneity of their responses. Finally, although the study provides valuable insights into the experience of uncertainty among nursing professionals during the Covid-19 pandemic, the constantly changing dynamics of the pandemic situation suggest a need for deeper exploration of issues involving post-Covid illness sequelae. Given the numerous vulnerabilities presented by the interviewees, it is clear that merely curing the disease biologically is not enough to address the impacts on these professionals’ mental health.

### Contributions to the Field

This study is significant as it utilizes a nursing theory to analyze and discuss a phenomenon that directly interfered with both the work process and the lives of professionals in this field, who represent the largest workforce in both Brazilian and global health services.

## FINAL CONSIDERATIONS

From the perspective of Merle Mishel’s antecedents of disease, the illness of nursing professionals from Covid-19 demonstrated that before being professionals, they are human beings like everyone else.

In the context of a global public calamity, it became clear that the antecedents of the disease strongly influenced the appraisal of uncertainty in the illness of nursing professionals from Covid-19, with significant emotional impacts on their lives. Media coverage of the increase in cases worldwide, the collapse of the healthcare system, and the high number of deaths contributed to the perception of the disease being associated with fear and panic in the face of potential illness, thereby intensifying the sensation of threat.

Given the many vulnerabilities presented by these individuals, and considering that curing the disease alone does not transform uncertainty into a new positive sense of order, it is necessary to develop a care plan for these professionals in their workplaces.
