# Supplementary material for: Uncertainties experienced by nursing professionals who contracted Covid-19: a priori, everyone is human
Source: Rev Bras Enferm. 2024 Jun 28;77(Suppl 1):e20230142. doi: 10.1590/0034-7167-2023-0142 (PMC11213537; doi:10.1590/0034-7167-2023-0142)
Supplement: Translation

# Translation: Incertezas vivenciadas por profissionais de enfermagem que adoeceram pela Covid-19: a *priori*, todos são humanos

**DOI:** 10.1590/0034-7167-2023-0142pt

**Authors:** Cinthia Rafaela Amaro Gonçalves Andrade, Laís de Miranda Crispim Costa, Ingrid Martins Leite Lúcio, Janaina Ferro Pereira, Herika do Nascimento Lima, Renata Lira do Nascimento

**Affiliations:** I Universidade Federal de Alagoas. Maceió, Alagoas, Brasil

**Keywords:** Enfermagem, Teoria de Enfermagem, Profissionais de Enfermagem, Covid-19, Infecções por Coronavírus

## Abstract

**Objetivo:**

Analisar as incertezas vivenciadas por profissionais de enfermagem que adoeceram pela Covid-19.

**Métodos:**

Pesquisa qualitativa realizada com 20 profissionais de enfermagem que adoeceram pela Covid-19. A coleta de dados ocorreu por meio de entrevista semi-estruturada; os dados foram organizados a partir da análise temática e discutidos à luz da Teoria Reconceptualizada da Incerteza da Doença de Merle Mishel.

**Resultados:**

Os antecedentes da doença tiveram forte influência na apreciação da incerteza dos profissionais de enfermagem que adoeceram pela Covid-19. A veiculação midiática do aumento de casos, o colapso do sistema de saúde e o grande número de mortes contribuíram para que a percepção da doença fosse associada ao medo e ao pânico.

**Considerações Finais:**

Na perspectiva dos antecedentes da doença, o adoecimento do profissional de enfermagem pela Covid-19 mostrou que, antes de ser profissional, ele é um ser humano como qualquer outro, passando por adversidades e potencialidades de pessoas adoecidas.

## INTRODUÇÃO

Este estudo teve como objeto a experiência de incerteza vivenciada por profissionais de enfermagem durante o processo de adoecimento pela Covid-19. Causada pelo coronavírus SARS-CoV-2, a Covid-19 é uma doença respiratória aguda, potencialmente grave em seres humanos, que possui altas taxas de contaminação e disseminação mundial^(1)^. O conhecimento sobre a transmissão da Covid-19 vem sendo continuamente atualizado. Sabe-se hoje que o período de incubação é estimado entre 1 e 14 dias, com mediana de 5 a 6 dias. A principal forma de transmissão é de pessoa para pessoa através de gotículas respiratórias. A maioria das transmissões ocorre de pessoas sintomáticas para outras, no entanto, estudos indicam que cerca de 45% de toda a transmissão ocorre antes do início dos sintomas^(2)^.

A pandemia da Covid-19 e suas repercussões vão muito além do campo biológico e da corrida tecnicista da ciência em busca de medidas de controle, tratamento, vacinas e de produção de conhecimento para controlar a expansão do vírus. Também reverberou nas dimensões econômica, psíquica e social, as quais implicam nos determinantes e condicionantes de saúde, não apenas nos adoecidos, mas na população em geral^(3)^.

“A pandemia compõe-se de inumeráveis surtos e epidemias, dores individuais e familiares, semelhantes nos aspectos biológicos e incomparáveis em outros sentidos”^(4)^. Pelo exposto, o estudo faz-se relevante pelas contribuições científicas no que tange ao adoecimento por profissionais de enfermagem acometidos pela Covid-19 à luz do conhecimento próprio dessa área do conhecimento.

## OBJETIVO

Analisar as incertezas vivenciadas por profissionais de enfermagem que adoeceram pela Covid-19.

## MÉTODOS

### Aspectos Éticos

O estudo foi conduzido de acordo com as diretrizes de ética nacionais e internacionais e aprovado pelo Comitê de Ética em Pesquisa da Universidade Federal de Alagoas, cujo parecer foi anexado durante o processo de submissão. Previamente às entrevistas, os participantes leram e assinaram o Termo de Consentimento Livre e Esclarecido. No sentido de assegurar o anonimato, os participantes receberam uma codificação abreviada: “ENF” para enfermeiros e “TEC ENF” para técnicos de enfermagem. Assim, a codificação alfanumérica obedeceu à categoria profissional seguida do número da ordem de realização das entrevistas.

Foram respeitadas as diretrizes e normas éticas previstas na Resolução 466/12 e na nº 510/2016 do Conselho Nacional de Saúde sobre pesquisas que envolvem seres humanos.

### Tipo de Estudo

Estudo exploratório-descritivo de natureza qualitativa. Foi utilizado o instrumento COREQ (*Consolidated Criteria for Reporting Qualitative Research*).

### Cenário do Estudo

O estudo foi realizado no estado de Alagoas, situado ao leste do Nordeste brasileiro, que conta com 102 municípios e uma delimitação espacial de 27.778,506 Km^2^, com limites territoriais nos estados de Sergipe, Bahia, Pernambuco e com o Oceano Atlântico.

### Fonte de Dados

Participaram do estudo 20 profissionais de enfermagem, cujos depoimentos foram colhidos através de entrevista semi-estruturada. Os critérios de inclusão foram profissionais de enfermagem com diagnóstico confirmado de Covid-19 e que residiam no estado de Alagoas, no período de outubro de 2020 a maio de 2021, assim caracterizados: 16 enfermeiros e 4 técnicos de enfermagem, com faixa etária variando de 27 a 46 anos de idade, dos quais apenas 2 eram do sexo masculino. Quanto ao estado civil, 16 eram casados, 3 divorciados e 1 viúvo. A cor de pele referida como prevalente foi parda (14), seguida de 5 brancos e 1 preto. Os participantes possuíam alta carga horária de trabalho, com 11 deles referindo 100 horas/semana, e mais de um vínculo de trabalho.

A confidencialidade do estudo foi preservada, pois não houve divulgação pública de resultados que permitissem a identificação dos momes dos participantes, visto que foi realizada uma codificação alfanumérica.

### Coleta e Organização dos Dados

Após a aprovação pela Plataforma Brasil, realizou-se a aproximação com os participantes, iniciando com 03 profissionais de enfermagem que adoeceram por Covid-19, conhecidos das pesquisadoras. A partir de então, cada um desses 03 profissionais indicou mais 01, conforme os critérios de inclusão, e assim sucessivamente, de modo que a coleta só foi finalizada após ser verificada a saturação de dados^(5)^, que é atingida quando a ampliação de dados e informações não modifica a compreensão do fenômeno pesquisado. Os participantes foram abordados presencialmente ou por telefone, momento em que a pesquisadora apresentou a proposta do estudo e, mediante o aceite, agendou a entrevista. A coleta de dados ocorreu entre janeiro e julho de 2021.

As entrevistas foram conduzidas pela pesquisadora principal e duraram cerca de 20 minutos. Foram realizadas em local reservado, previamente acordado com o participante. Realizou-se a gravação dos encontros em gravador digital. O conteúdo das entrevistas foi transcrito na íntegra em documento do *Microsoft Word* para posterior análise.

### Análise dos Dados

Foi realizada a análise temática de Minayo^(5)^, que contempla três etapas: 1) pré-análise, a partir de leitura flutuante, constituição do corpus e (re)formulação de hipóteses e objetivos; 2) exploração do material; e 3) tratamento dos resultados obtidos e interpretação. Utilizou-se como referencial teórico a Teoria Reconceptualizada da Incerteza da Doença de Merle Mishel (TRID)^(6)^.

A TRID discorre que as incertezas ocorrem em todas as fases do adoecimento (antes, durante e depois). Este pensamento é consolidado a partir dos três pilares da teoria: 1) Antecedentes da doença; 2) Avaliação cognitiva da incerteza; e 3) Estratégias de enfrentamento - “*Coping*”^(6)^. Estas categorias subsidiaram a seleção dos discursos dos participantes que serão apresentados nos resultados. Ademais, ao longo da trajetória da doença, os indivíduos podem perceber aspectos benéficos da incerteza, de modo que ficam motivados a aproveitar cada momento de suas vidas, o que Mishel rotula de crescimento através da incerteza^(7)^.

## RESULTADOS

Como uma doença nova, a Covid-19 se apresentou como um dos maiores desafios sanitários em escala global deste século. Algumas falas mostram como essa realidade era desconhecida até então:

[...] *logo no início da pandemia, na nossa realidade, enquanto Brasil, foi um pouco assustadora. Não foi fácil ver as pessoas no Equador serem carregadas por caminhões. Corpos sendo carregados e em alguns lugares não se tinha mais onde enterrar* (ENF 01)

[...] *o cenário já estava estourando em alguns países e não imaginava que chegaria tão devastador no Brasil. Mas foi um momento de tudo novo. Tudo vago.* (ENF 03)

[...] *o início da Pandemia em março de 2020 trouxe uma série de incertezas para todos de uma forma geral. Não sabíamos o que iríamos enfrentar* (ENF 09).

[...] *era uma questão nova, pandemia, um vírus que ninguém conhecia, que ninguém sabia que poderia acontecer.* (ENF 18)

[...] *na verdade, quando as pessoas começaram a falar sobre a Covid, eu não me interessei muito porque achava que ela não chegaria até aqui no Brasil.* (ENF 10)

[...] *embora já se falasse em Covid, eu não acreditava que aquilo ia acontecer aqui.* (ENF 20)

[...] *quando eu estava vendo na televisão parecia que não era real. Quando vem para perto de você, que é dentro do seu trabalho, aí você olha assim, meu Deus é real. Entendeu? Eu só comecei a perceber a situação quando chegou aqui. Mas antes, era irreal.* (ENF 02)

Analisando o contexto geral, a Covid-19 é um fenômeno de importante magnitude e relevância, cuja informação foi disseminada pelas mídias sociais e pelo jornalismo:

[...] *fiquei bastante preocupado por ter visto nos veículos de comunicação a gravidade da doença no organismo do ser humano e isso me abalou muito.* (ENF 09)

[...] *antes de chegar essa doença aqui no Brasil, eu assistia muito jornais. Quando eu via as notícias na televisão, eu ficava nervoso. Eu via na Itália, Espanha, nos países ricos, muita gente morrendo.* (TEC ENF 04)

[...] e*ntão eu evitei muito ver reportagens. Essa parte assim: “Ah, todo dia morre alguém”.* (ENF 03)

Apontaram, ainda, que no início da pandemia existia uma escassez de informações e de protocolos padronizados para o manejo clínico e práticas baseadas em evidências científicas, não sendo possível fazer julgamentos claros:

[...] *como era uma doença muito nova, tinha muita informação desencontrada.* (ENF 14)

[...] *naquela época, havia pouco conhecimento sobre Covid-19, ou seja, era uma coisa nova.* (ENF 20)

[...] *e eu só sabia as informações de fora. Sabia que muitas coisas estavam fechando e com relação ao tratamento deveria manter sempre o isolamento.* (ENF 03)

[...] *depois veio o “boom”, veio o uso das máscaras porque era uma doença transmitida pelo ar, então naquele momento que adoeci meu conhecimento era esse.* (ENF 11)

No início da pandemia, como as informações de saúde ainda estavam sendo processadas/construídas, os entrevistados apontaram que as mudanças constantes nas informações não permitiam o processamento destas e os expunham a uma fragilidade no conhecimento:

[...] *eu acho que não consigo trazer como conhecimento o que estava passando nos jornais naquele momento porque as informações que estavam acontecendo ao redor do mundo eram coisas muito pontuais e ainda não se tinha definido o que era.* (ENF 01)

[...] *chegou para gente como muita coisa na nossa cabeça, que a gente não sabia até onde ia, até onde não ia, como era. Eu sei que chegou no exagero.* (ENF 02)

[...] *no começo por ser uma doença nova, existiam muitas informações desencontradas.* (ENF 09)

Assim, o contexto de insegurança por uma doença nova, com tantos desencontros de informações, fomentou o espanto e amedrontamento diante da possibilidade de adoecimento, gerando incertezas antes mesmo do fato ocorrer. Nestas circunstâncias, enfatiza-se que o fato de ser profissional da área de saúde não retira deste trabalhador a essência do *“Ser humano”*, “*Ser sujeito*”.

[...] *o que a TV mostrava eram os casos graves, eu fiquei com muito medo de adquirir uma Síndrome Respiratória aguda grave. É mais o medo em torno da doença.* (ENF 10)

[...] *a verdade era essa, você só via os casos aumentando, você só via as pessoas piorando, você só via todo mundo pegando. Espera com receio de chegar a sua vez.* (ENF 16)

[...] *você não podia pegar em nada, você não podia pegar em dinheiro, você não podia pegar em mesa, não podia pegar em bancada, em todo canto você podia adquirir a doença.* (ENF 03)

[...] *me manter afastada de tudo, principalmente do trabalho, visto que sou muito ativa e dinâmica, também foi complicado.* (ENF 06)

Em consonância com este pensamento, os profissionais de enfermagem que adoeceram pela Covid-19 no início da pandemia apresentaram falta de clareza quanto à doença pela escassez de informações.

[...] *o que eu entendia por Covid era que era uma doença muito contagiosa, respiratória, que os sintomas iniciais eram febre, uma fraqueza e até então só.* (ENF 11)

[...] *até o momento eu sabia que era grave, mas não era algo que eu tivesse tanto conhecimento até pela falta de informações no geral.* (TÉC ENF 07)

[...] *o que é que leva um vírus a ter um agravamento tão forte com uma sintomatologia inicial tão pequena, tão desprezível até e ter uma gravidade tão grande, o que é que leva isso a acontecer de pessoa para pessoa? Nós sabemos que tem pessoas que se comportam muito bem e tem outras que chegam à morte.* (ENF 08)

[...] *“você tem conhecimento de tudo?” Não! Não tenho, porque cada dia muda! Ontem mesmo eu assisti uma live, com um cardiologista e um infectologista, dizendo coisas que me deixou mais perdida do que antes! É cada dia uma novidade!* (ENF 12)

Em alguns momentos, a produção científica foi crucial para melhor aproximação da compreensão sobre a doença, seus efeitos e a busca por soluções, como podemos observar a seguir:

[...] *eu lembro que muitos manuais eles foram feitos várias versões, então a gente estava sempre acompanhando, este processo que foi bem dinâmico, mas eu acredito que absorver esse conhecimento do Ministério da Saúde foi em conjunto com o processo que era construído na secretaria de saúde.* (ENF 01)

[...] *de certa forma, os pesquisadores estão aprimorando a cada dia as evidências científicas sobre a doença, melhorando a informação para os profissionais de saúde e a população no geral.* (ENF 09)

[...] *foi muito importante o grau de conhecimento, de informações e tratamento que a gente conseguiu evoluir de 2020 para cá. Aos poucos o conhecimento está ficando mais consolidado.* (ENF 14)

[...] *mesmo não sabendo tanto, mas a gente já sabe que as pessoas que estão no grupo de risco são as pessoas mais propensas a se agravar.* (ENF 19)

No entanto, com o aparecimento de casos que fugiam do perfil das pessoas do grupo de risco, o medo era acentuado:

[...] *a realidade de hoje nos mostra que não existe grupo de risco não, porque desde o RN até o idoso está havendo mortalidade, então acabou, tirou por terra essa história de quem tem doença grave, comorbidades. Isso aí caiu por terra, não adianta.* (ENF 01)

[...] *nós sabemos que pessoas com muito conhecimento que estão tendo todos os cuidados, que têm uma vida boa, relativamente assim em termos de alimentos, em termos de cuidados pessoais e tudo mais, que não são sedentários etc etc e que estão desenvolvendo casos gravíssimos e até falecendo, contradizendo tudo que inicialmente foi dito.* (ENF 08)

[...] *em cada pessoa ela reage de uma forma diferente. Porque é uma questão de organismo de cada pessoa. Tem pessoas que por exemplo, que vai verificar uma saturação de oxigênio está em 97%, de repente cai para 93%. Tem pessoas que satura 97, 98 e sente dispneia e sente falta de ar, fadiga, que é um sono, um sono que dá muito grande.* (ENF 13)

[...] *eu perdi muitos entes queridos, perdi agora, tem umas duas semanas, perdi um primo de 48 anos que não tinha nenhuma comorbidade, era até ciclista, era uma pessoa que era bem saudável mesmo, mas infelizmente não teve a mesma sorte que eu. A gente nunca vai esquecer essa parte aí que é bem complicada também né, perder os entes queridos, mas é assim mesmo.* (TÉC ENF 15)

[...] *assim, a gente já sabia que era uma doença grave, todo mundo estava com risco.* (TÉC ENF 05)

Também foi relatado pelos participantes o medo do adoecimento pela Covid-19, apontando insegurança diante de um possível diagnóstico da doença:

[...] *olhe, na verdade o grande desconforto é que a gente não sabe o que é que vai acontecer. Então a insegurança. A gente sente uma insegurança porque nada é fechado, não existe uma fala fechada, né?! (*ENF 08)

[...] *hoje, mesmo com a vacinação e mesmo estando vacinado e já ter adoecido, o medo em mim é latente. Sabemos que esse mal ainda é novo, e existe um longo caminho a se percorrer para garantir que um fármaco possa auxiliar e combater o vírus no organismo do ser humano.* (ENF 09)

Nesse contexto, o medo de morrer pela Covid-19 atingiu alguns participantes:

[...] *o conhecimento que a gente tinha era um conhecimento que até todo mundo achava que não sabia se estava certo, se estava errado, porque era uma doença que no final mesmo você pensava assim “ah todo mundo vai pegar e todo mundo vai morrer, não vai sobrar ninguém no mundo”.* (ENF 16)

[...] *como estava na linha de frente, fiquei muito tempo cuidando de pacientes com comprometimento pulmonar de cinquenta, setenta e cinco, oitenta por cento, então eu fiquei com medo. Foi a primeira vez que eu fiquei com medo de morrer na vida.* (ENF 13)

[...] *não ansiedade… o medo, porque eu já tinha infartado, então eu tinha medo de morrer.* (ENF 12)

O medo de contaminação determinou a implementação de cuidados preventivos de higiene, proteção individual e rotina de limpeza em congruência com as orientações feitas pela Organização Mundial de Saúde:

[...] s*aber que a gente tem que melhorar os nossos hábitos de higiene, a nossa etiqueta respiratória, que a gente falou tanto de etiqueta respiratória esse ano, não é?* (ENF 01)

[...] *utilizei as armas que eu tinha contra o Coronavírus. Desde o começo da pandemia fiz o isolamento social, o uso das máscaras, uso do álcool em gel com frequência para tentar de alguma forma afastar essa doença da minha pessoa.* (ENF 09)

[...] *então foi mais ou menos isso, essa precaução, lavar muito a mão, passar álcool, evitar está apoiada nos móveis.* (TEC ENF 05)

[...] *mas assim, a importância da muda de roupa quando chega em casa, de evitar a entrada pela porta principal de ter uma entrada alternativa, eu acho que é alguns cuidados que a gente foi tendo, foi aprendendo para lidar com esta doença específica.* (ENF 02)

[...] *o que é que a gente poderia fazer!? Que foi manter o distanciamento social, não receber visitas em casa. Foi uma opção da gente, quando os bebês nasceram, não tá recebendo essas visitas de familiares, parentes e tudo.* (ENF 14)

Neste contexto, reforça-se o pensamento de que a influência dos antecedentes da doença, como a atuação na linha de frente no enfrentamento à Covid-19, adoecimento de familiares, amigos e pessoas próximas, bem como adoecimentos anteriores, condiciona a incerteza ao pensamento probabilístico e à busca pela previsibilidade dos acontecimentos:

[...] *então, eu fui diagnosticada com Covid em junho de 2021. Mas, eu comecei a apresentar alguns sintomas no início de maio, que é até estranho o intervalo de tempo para dizer que eu adoeci em maio por Covid.* (ENF 19)

[...] *acho que quando a gente é da parte da saúde acho que você já espera o que vai acontecer com a gente.* (TÉC ENF 05)

[...] *justamente por estar na área a gente sabe da gravidade da doença.* (ENF 17)

[...] *um belo dia a minha mãe disse para mim, minha mãe tinha 83 anos, ela disse para mim: minha garganta está arranhando. Eu disse como a garganta está arranhando? Aí a gente já ficou preocupado. Tudo poderia ser Covid, não é!?* (ENF 20)

[...] *eu acho que a gente piora mais ainda porque a gente não só sabe, mas a gente tem a vivência do dia a dia dos pacientes que pegaram e que realmente ficaram graves.* (ENF 16)

O apoio social da família está entre os provedores de estrutura que auxiliam o indivíduo na apreciação da incerteza. Ainda assim, alguns profissionais optaram em afastar-se dos familiares por atuarem na linha de frente contra a Covid-19 como forma de proteção dos seus:

[...] *então a partir do momento que eu decidi trabalhar com Covid eu saí da minha casa e evitei a minha família por alguns meses.* (ENF 19)

[...] *quando eu iniciei, comecei a trabalhar diretamente com Covid eu decidi sair de casa, para prevenir a minha família já que eles estavam cumprindo todo o distanciamento social. Então eu preferi sair e manter eles protegidos, que foi um benefício.* (ENF 03)

[...] *é questão pessoal, eu tenho pessoas idosas na família, então tive cuidado de não estar visitando, não estar aglomerando. Porque a gente sabia que poderia, estando em ambiente hospitalar, passar para alguma pessoa da família que estivesse mais vulnerável. Então eu tive este cuidado e até hoje tenho. Mas assim, não é fácil.* (ENF 13)

As mudanças nas relações interpessoais cotidianas, após o surgimento da Covid-19, foram apresentadas com fala marcante na condicionalidade das relações por recursos digitais e de telecomunicação, fragilizando os vínculos familiares e de amizade:

[...] *passei mais de cinco meses sem poder visitar minha mãe, minha sobrinha, meu irmão e meu cachorro. Essa sensação traz um sentimento de impotência de como nós seres humanos somos dependemos de carinho, afeto e amor* [...] *falar via telefone, nunca vai ser a mesma coisa que pessoalmente. Mandar uma foto por telefone, nunca vai ser a mesma coisa que um abraço ou beijo no rosto, ou um carinho do seu cachorro, ou encontrar com seus amigos de infância no local em que você cresceu.* (ENF 09)

As experiências prévias de adoecimentos por outras doenças que levaram a um agravamento do quadro também foram apresentadas como antecedentes da doença, que despertam incertezas antes mesmo do adoecimento por associação ao quadro de exacerbação anterior:

[...] *ficar internado, isso faz com que os fantasmas da minha infância venham à tona, até porque meu irmão ficou mais de 25 dias internado em um leito hospitalar para tratar a Covid-19, tendo mais de 50% dos pulmões comprometidos.* (ENF 09)

Ainda sobre as experiências de vida dos participantes, os eventos de memória ligados a parentes e amigos próximos foram citados fomentando a familiaridade dos fatos.

[...] *eu lembro que nessa Pandemia a mãe de um amigo meu faleceu e assim não foi fácil para ele e assim eu lembro de um momento da gente ter ficado muito pensativo sobre isso pelo fato da gente se imaginar nessas situações. De se imaginar na situação de adoecer quem a gente ama e perder quem a gente ama. De ser o vetor da doença.* (ENF 01)

[...] *no dia seguinte meu irmão estava piorando e ele foi encaminhado para ser internado. Ele se recuperou e mesmo no leito do hospital, ele pediu para eu me cuidar ainda mais porque a doença não é fácil. Tive sentimento de impotência quando meu irmão e minha prima estiveram internados, foi o mesmo sentimento quando meu pai também ficou internado e veio a falecer meses depois.* (ENF 09)

[...] *graças a Deus, os meus foram leves, mas, por exemplo, eu tive uma tia que ficou internada quatorze dias. Não precisou ser entubada, nem nada, mas estava lá no oxigênio. Então, a gente tenta passar essa orientação para família, para os amigos mais próximos, que estão sempre procurando saber como fazer.* (ENF 14)

Desta forma, o profissional de enfermagem faz uso da experiência do núcleo social como recurso para interpretação dos estímulos de incerteza.

[...] *mas quando chegou aqui, olhando, vendo as pessoas, ouvindo: “Fulano vai morrer!”, então está na sua frente* [...] *o cenário se agravando. Os colegas ficaram na UTI. Colegas que não conseguiram sair ainda, que está no SARA tentando se reerguer, caminhar, falar direito. Tudo isso mexe com a gente. Então é complicado.* (ENF 02)

[...] *sei que assim, mesmo tendo visto tudo isso, nos dois serviços eu lembro bem que eu não surtei. Mas eu tive amigas que ficaram internadas, eu tive amigas que perderam familiares e a gente, quer queira quer não fica assim, mas eu não surtei ao ponto de dizer assim. Deu para trabalhar com equilíbrio.* (ENF 03)

[...] *tive colegas de trabalho, fisioterapeuta, médico, enfermeiro, técnico, ficar grave mesmo e a gente ficava naquela loucura “meu Deus, meu Deus, tem que sair, tem que sair, tem que ficar bem” e era uma luta diária e assim… piora muito mais, eu acho que a gente ficava mais angustiada, mais nervosa, mais pensativa, no fato de você ter a vivência do dia a dia.* (ENF 16)

Antecendentes da saúde mental fragilizada são elencados como fatores que intensificam a condição de fragilidade apresentada.

[...] *precisei de ajuda. Precisei me afastar. Precisei ficar cinco meses afastada e eu precisei me ajudar.* (ENF 02)

[...] *eu acho que era mais psicológico, devido o infarto. Mas eu tive síndrome do pânico.* (ENF 12)

Na linha de frente no combate ao novo coronavírus, os profissionais de enfermagem, assim como os médicos e tantos outros profissionais, conviveram diariamente com um cenário de duplicidade de incertezas, tanto na execução do cuidado como no adoecimento. Isso causou estresse e preocupação para a rotina desses profissionais:

[...] *eu trabalho em UTI, então é um momento que você precisa ter sangue no olho. Precisa ter um pouco de sangue frio. Porque? Porque você precisa ter técnica para agir, para trabalhar e você precisa ser rápido. E nem todo mundo tem essas características. Então o que muitas vezes eu vi acontecer, diversas e diversas vezes, foi o fato de alguém se sujar com secreção ou sangue, ou secreção traqueal, seja qual for, e achar que tinha se contaminado e achar que ia adoecer, e não! A gente precisa ter calma.* (ENF 01)

[...] *começou que os pacientes, com muita rapidez, descompensavam. Pacientes entrando em parada cardiorrespiratória. Pacientes indo a óbito em uma fração de tempo. Por exemplo, paciente dava entrada em uma unidade hospitalar hoje, daqui a três, quatro dias estava intubado. Daqui a pouco o paciente começava a usar todas as drogas que estão ao alcance em termo de vasoativas para manter aquele paciente. De repente teria uma complicação. Paciente teria que dialisar. E de repente o paciente vem a óbito. Uma semana, às vezes menos. Muito rápido.* (ENF 13)

[...] *porque assim, eu tenho uma amiga no trabalho, que não é aqui, eu fico olhando e respeito, mas ela chega, ela coloca um plástico na cama onde ela descansa, ela joga Lysoform [produto de limpeza bactericida] na cama, e dorme em um saco. Ela descansa em um saco. Toda embrulhadinha em um saco. Assim, você vê que chegou no limite do estresse. Esse é o limite do estresse.* (ENF 02)

## DISCUSSÃO

No presente estudo, evidenciamos a presença marcante de relatos associados aos antecedentes vividos antes do adoecimento do profissional de enfermagem pela Covid-19. Observa-se que estes possuem influência direta na apreciação da incerteza. Conforme desvelado na pesquisa, nos antecedentes da incerteza, encontramos incentivo, encorajamento, estímulo, impulso, instigação, entre outros tantos adjetivos que propulsionam as capacidades cognitivas e os mecanismos que funcionam como fontes de estímulo para a incerteza.

Cabe apontar que o contexto da pandemia trouxe algumas peculiaridades quanto ao processo de adoecimento, visto que era esperada a contaminação em massa da população. Entre os adoecidos, estavam os profissionais de saúde, em especial os de enfermagem, que, como seres humanos, carregam uma história de vida e uma percepção do mundo que os cercam. Sendo assim, experiências novas passam pelo crivo de avaliação associada às experiências prévias.

Para Mishel, os antecedentes da doença conferem ou possibilitam à pessoa a capacidade para desencadear o processo de apreciação e a tomada de decisão. Na vivência do adoecimento por Covid-19, o ponto de partida abala essa experiência, uma vez que a fragilidade experimentada condiciona a capacidade cognitiva e, consequentemente, o processamento da informação^(6)^.

No cuidado de enfermagem à pessoa adoecida pela Covid-19, a atuação na linha de frente também traz implicações no campo das representações dos sentimentos, entre eles: solidão, desamparo, cansaço físico, estresse, irritabilidade, nervosismo e esgotamento mental. É importante destacar que tais sentimentos impactam de forma negativa nas relações humanas e nas práticas sociais dos sujeitos, em especial nas de cuidado. Logo, “podem comprometer a capacidade para tomar decisões, pelo medo, incapacidade de enfrentar o sofrimento, falta de conhecimento”^(8)^.

Esse conjunto de elementos evidencia que o cenário pandêmico instalado estava coberto por incertezas, com repercussões individuais, permeadas de sentimentos, desafios, dificuldades, imagens e comportamentos próprios de cada indivíduo. Assim, evidências científicas apontam que os antecedentes são fundamentais para a compreensão das experiências individuais nas situações de doença, já que esta pode afetar a compreensão sobre o processo de adoecimento, os resultados da evolução da doença, bem como as respostas adaptativas^(6-9)^.

O componente Antecedente da Doença, como apresentado por Merle Mishel em sua TRID, pode ser discutido elucidando o que cada pessoa traz para a vivência da incerteza, face ao processo de transição saúde-doença, que influencia no modo como a pessoa acolhe a incerteza e lida com ela. Nos antecedentes da doença encontramos a motivação, as capacidades cognitivas e os mecanismos que funcionam como estímulo para a incerteza^(6-7)^.

No contexto estudado, a mídia também representou uma importante influência no grau de incerteza gerada, atuando como fonte de estímulo da incerteza antecedente ao fenômeno, com desfecho de um efeito negativo de ameaça diante do adoecimento pelo alto impacto das informações noticiadas. Nessas situações, em que as pessoas lidam com o pouco conhecimento ou a falta de entendimento sobre o que vivenciam, manifestam-se em um comportamento de busca de informações que, por vezes, são instáveis e/ou inconsistentes^(6-9)^, visto que as evidências científicas de manejo clínico e afins ainda estavam em processo de construção/consolidação.

Pelos relatos, percebe-se um discurso generalizado de pessoas que buscavam conhecimentos básicos de saúde, apesar de serem profissionais da área. O progresso terapêutico e os aspectos práticos de como lidar com as demandas diárias e contínuas do cuidado de si são possíveis com a apreciação da condição de saúde e as vulnerabilidades associadas^(6-9)^. Houve um medo extremo de contrair a infecção^(10)^, cuja semelhança dos sintomas psicológicos nos profissionais de saúde é comparável com outros surtos, como o de SARS e o de ebola^(11)^.

Na perspectiva da TRID, as incertezas geradas nas experiências de doença englobam ideias de fragilidade na informação, dúvidas, probabilidades, incapacidades, desconforto e insegurança nas resoluções, sensações difíceis e sentimentos de controle e autoconfiança. Assim, explica-se a singularidade das respostas dos indivíduos a partir de experiências e vivências anteriores, definindo futuras possibilidades de existência^(12)^.

Também foi possível perceber que as incertezas repercutem como inadequação no estado cognitivo, prejudicando a interpretação e avaliação dos acontecimentos da vivência da doença e comprometendo a adaptação da pessoa adoecida. Para Mishel, a falta de informação ocorre quando o compartilhamento destas informações sobre a doença é ausente ou insuficiente, necessitando da adição de instrução a respeito para a construção de um conhecimento fundamentado^(6-7)^.

À medida que as informações iam sendo validadas, estas eram atualizadas e publicadas de forma dinâmica em curtos intervalos de tempo. As inseguranças, nesse sentido, podem ser amenizadas com o fornecimento de informações sobre as causas e consequências dos sintomas. Na conjuntura da Covid-19, os profissionais não tinham conhecimento de causa e os cuidados à pessoa adoecida ainda não faziam parte da rotina de trabalho^(13)^.

Nesse ínterim, as capacidades cognitivas são atributos que os indivíduos possuem para a averiguação de determinados dados e informações. Esta capacidade se desvela tanto por respostas inatas como por respostas específicas a determinadas situações. A inferência, por sua vez, refere-se à avaliação da incerteza, utilizando as experiências prévias para o processamento da vivência atual, estabelecendo familiaridade com os fatos. É o julgamento que o indivíduo faz diante dos fatos vivenciados^(6-7)^.

Sobre este aspecto, um estudo qualitativo com 21 enfermeiros que trabalharam na unidade de emergência ou terapia intensiva em um hospital da Turquia durante a pandemia evidenciou que, apesar das múltiplas fontes de incerteza vivenciadas pelos participantes, estes profissionais procuraram focar no cuidado ao paciente e na satisfação como forma de gerenciar a incerteza e conferir sentido à sua experiência, reconhecendo inclusive seu crescimento pessoal e profissional durante todo o processo^(14)^.

Cabe destacar que, pela atipicidade do contexto da pandemia, os antecedentes da doença já se apresentavam cercados de incertezas e já faziam parte do processo de adoecimento pelo receio do acometimento. Este, quando somado à vulnerabilidade do pertencimento ao grupo de risco, com maior propensão ao agravamento do quadro de saúde, acentuava o temor. Para os participantes que pertenciam à faixa de maior vulnerabilidade às complicações causadas pela Covid-19, a sensação de ansiedade se apresentou com mais relevância. Para Mishel, o impedimento do controle frente à situação gera dificuldade de ordenamento acerca do que se conhece sobre a doença, favorecendo o sentimento de insegurança^(6-7)^.

A insuficiência em conferir valor ao processo de adoecimento está diretamente relacionada à incerteza. Estudos indicam que a instalação da incerteza frente ao adoecimento acarreta em uma desordem nos sentimentos e atitudes, levando ao afastamento do indivíduo de seu núcleo social, gerando desesperança, falta de auto reconhecimento e descaracterização da personalidade. Portanto, a teoria evidencia que é imprescindível a reorganização e reestruturação das estratégias de enfrentamento a partir da manifestação da incerteza^(6-7)^.

Os trabalhadores da enfermagem envolvidos no cuidado aos pacientes com Covid-19 se expuseram sobremaneira ao risco do adoecimento, sendo que “a heterogeneidade que caracteriza este contingente da força de trabalho determina formas diferentes de exposição, tanto ao risco de contaminação quanto aos fatores associados às condições de trabalho”^(15)^.

Apesar dos profissionais de saúde estarem acostumados a lidar com situações de tensão psicológica no trabalho, a pandemia de Covid-19 trouxe uma situação diferente, visto que precisaram lidar com o medo de se contaminar e de expor seus familiares ao risco. Calcado em experiência prévia, os antecedentes da doença permitem uma análise probabilística do adoecimento quanto ao julgamento enquanto ameaça ou oportunidade. Condicionam o grau de adaptação à familiaridade dos fatos e às vivências prévias, sendo estas evidenciadas mais claramente nas falas de profissionais que atuaram na linha de frente da assistência às pessoas acometidas pela Covid-19.

Assim, os profissionais envolvidos no enfrentamento da pandemia experienciaram as mais diversas evoluções de casos (leves, moderados, graves e críticos) e prognósticos, tomando para si o significado do adoecimento a partir de suas vivências. Para tanto, as associações entre os sinais e sintomas dos pacientes, na prática clínica, durante o exercício de sua atuação profissional, foram realizadas no sentido de estabelecer similaridade e familiaridade às condições de saúde em busca de suporte palpável e certeza para consolidar o conhecimento a respeito da doença.

Segundo Merle Mishel, a redução da incerteza ocorre diretamente ligada aos provedores de estrutura. Estes ajudam o indivíduo a determinar o padrão de sintomas, a relação dos eventos e a congruência das experiências, conforme aumentam o padrão de sintomas, esses três aspectos também se elevam^(6-7)^.

É notório que mudanças sociais e culturais, em função da morte e do adoecimento de pessoas ligadas afetivamente, deflagram a necessidade de reestruturação de posicionamentos, desempenho de papéis e de funções no núcleo familiar^(6-7)^. Relatos de lembranças e acontecimentos de adoecimento pela Covid-19 por familiares e pessoas próximas foram bastante evidentes.

Este recurso faz parte dos antecedentes da doença, associado aos estímulos relativos à doença e relacionados a questões fisiológicas, tais como a frequência, intensidade, quantidade, localização e duração; e à capacidade cognitiva relacionada às habilidades pessoais de processamento de informações^(6-9)^. Os eventos de memória com colegas de trabalho também foram evidenciados, de modo que, quando associados ao adoecimento pela Covid-19, que já traz muitas incertezas, potencializam a angústia, a desesperança na cura e conferem bloqueio nas estratégias de enfrentamento para otimização das respostas adaptativas.

Os profissionais de saúde estão expostos a uma alta carga e, pela natureza de seu trabalho, fazem parte do grupo de risco para contrair a Covid-19^(15)^. Além disso, estes profissionais passaram por situações de pressão, risco de infecção, excesso de trabalho, frustração, discriminação, isolamento, assistência a pacientes com emoções negativas, falta de contato com a família e exaustão.

Essas situações causam problemas de saúde mental, como estresse, ansiedade, ocorrência de sintomas depressivos, insônia, negação, raiva e medo. Problemas que afetam a atuação dos profissionais e também corroboram para as incertezas quando acometidos pelo adoecimento por Covid-19. Os eventos que causam a incerteza podem ser a maior fonte de estresse, provocando reações fisiológicas e aumentando as emoções^(6-7)^.

### Limitações do Estudo

Este estudo apresenta algumas limitações que devem ser consideradas na interpretação dos resultados. Primeiramente, a natureza qualitativa da pesquisa limita a generalização das conclusões para toda a população de profissionais de enfermagem do Brasil, visto que as experiências e percepções são intrinsecamente subjetivas e específicas ao contexto dos participantes. Outra limitação importante é a possibilidade de viés de seleção, já que os participantes foram escolhidos por meio de uma abordagem de amostragem em cadeia, o que pode ter influenciado a homogeneidade das respostas. Por fim, embora o estudo forneça achados valiosos sobre a experiência de incerteza entre os profissionais de enfermagem durante a pandemia de Covid-19, a dinâmica em constante mudança da situação pandêmica sugere a necessidade de aprofundamento das questões que envolvem as sequelas do adoecimento pós-Covid, pois diante de tantas fragilidades apresentadas pelos entrevistados fica evidente que apenas a cura biológica da doença não dá conta de ressignificar as reflexos na saúde mental destes profissionais.

### Contribuições para a Área

Este estudo é relevante pois utiliza uma teoria de enfermagem para analisar e discutir um fenômeno que interferiu diretamente no processo de trabalho e de vida dos profissionais dessa área, os quais representam a maior força de trabalho no âmbito dos serviços de saúde brasileiros e mundial.

## CONSIDERAÇÕES FINAIS

Na perspectiva dos antecedentes da doença de Merle Mishel, o adoecimento do profissional da enfermagem pela Covid-19 mostrou que antes de ser profissional ele é um ser humano como qualquer pessoa.

Diante de um contexto de calamidade pública no mundo, ficou evidente que os antecedentes da doença tiveram forte influência na apreciação da incerteza diante do adoecimento de profissionais de enfermagem pela Covid-19, com impactos emocionais signficativos na vida dessas pessoas. A veiculação midiática do aumento de casos pelo mundo, o colapso do sistema de saúde e o grande número de mortos contribuiu para que a percepção da doença fosse associada ao medo e pânico diante de um possível adoecimento, acentuando, por sua vez, a sensação de ameaça.

Diante de tantas fragilidades apresentadas por estes sujeitos e partindo do pressuposto de que a cura da doença por si só não ressignifica a incerteza em um novo sentido de ordem positivo, faz-se necessário a construção de um plano de cuidados para esses profissionais em seus espaços de trabalho.
